# Increased impact of GPIb receptors on platelet adhesion under flow in severe COVID-19

**DOI:** 10.52601/bpr.2025.250007

**Published:** 2026-04-30

**Authors:** Yuliya Avtaeva, Konstantin Guria, Ivan Melnikov, Anna Kalinskaya, Galina Artemyeva, Zufar Gabbasov

**Affiliations:** 1 Chazov National Medical Research Centre of Cardiology, Moscow 121552, Russia; 2 Institute of Biomedical Problems of the Russian Academy of Sciences, Moscow 123007, Russia; 3 I.V. Davydovsky Clinical City Hospital, Moscow 119027, Russia; 4 A.I. Evdokimov Moscow State University of Medicine and Dentistry, Moscow 127473, Russia

In recent years, coronavirus disease 2019 (COVID-19) has become a global challenge. Early on, during the pandemic, it became obvious that thrombotic complications are a major cause of morbidity and mortality in COVID-19. The subsequent use of anticoagulants in acute COVID-19 treatment has significantly improved the outcomes (Schulman *et al*. 2022). Nevertheless, pathological mechanisms causing COVID-19 associated coagulopathy (CAC) are still poorly understood due to their multifaceted nature. Investigation of these underlying mechanisms is of great importance as it may lead to further improvement of treatment strategies and therapeutic approaches (Page and Ariëns 2021).

One of the current concepts concerning the driving mechanisms of COVID-19 pathogenesis is the reciprocal mutual amplification of innate immunity and coagulation system (Conway *et al*. 2022). A combination of these processes is referred to as immunothrombosis and/or thromboinflammation, and it plays an important role in multiple clinical settings including acute respiratory distress syndrome and sepsis. One of the links between thrombotic and inflammatory processes is the von Willebrand Factor (vWF) known for promoting a prothrombotic state in inflammatory-induced conditions. A vast body of evidence indicates that vWF levels are increased multifold in COVID-19 patients (Favaloro *et al*. 2021). Moreover, an increased vWF concentration shows a strong correlation with disease severity and poor prognosis (Xu *et al*. 2022). At the same time, vWF-cleaving protease-ADAMTS-13 (a disintegrin and metalloproteinase with thrombospondin type 1 motif, member 13) activity shows a relative deficiency in severe COVID-19 patients (Favaloro *et al*. 2021). The decrease in ADAMTS-13 activity in COVID-19 patients is less radical than in the case of thrombotic thrombocytopenic purpura (TTP), but it still shows clear evidence for imbalance of the vWF-ADAMTS-13 axis in COVID-19 (Mancini *et al.* 2021; Joly *et al*. 2021).

vWF is a major player in primary haemostasis. In particular, it regulates platelet adhesion under flow. Damaged by SARS-CoV-2 endothelium can release ultra-large and high-molecular-weight multimers of vWF into the circulating blood. The decreased activity of ADAMTS-13 does not allow for the timely degradation of ultra-large vWF multimeters. Accumulated multimers can be unfolded and activated by mildly increased shear rates, exposing numerous binding sites for Glycoprotein Ib (GPIb) receptors of platelets. Interaction of GPIb with unfolded vWF multimers initiates platelet adhesion and subsequently activates multiple signaling pathways leading to further amplification of platelet adhesion and clot formation. Moreover, GPIb also interacts with leukocytes via their integrin CD11b/CD18 (Mac-1) amplifying even further clot growth and participating in so-called throboinflammation processes (Denorme *et al*. 2019).

Knowing the present imbalance of the vWF/ADAMTS-13 axis in COVID-19, one might expect platelet adhesion to be altered in such case as well. In this brief report, we present new data concerning platelet adhesion in severe COVID-19 patients. We also have analyzed the influence of GPIb on the adhesion process in COVID-19 patients. The main observation of our study is that, while platelet adhesion at high-shear rates is not altered much, the effect of the GPIb receptor on the adhesion process is substantially increased in COVID-19 patients.

A group of 17 consecutive patients with severe COVID-19 pneumonia admitted to the intensive care unit (ICU) from January to February of 2021 was included in this study. None of the patients were vaccinated. The study was approved by the Ethics Committee of the National Medical Research Centre of Cardiology. Written informed consent was obtained from the patient or the first next of kin. A group of healthy volunteers (*n* = 17) was used as a control. Healthy controls were not matched with patients with severe COVID-19. Platelet adhesion was assessed in a flow-through *in vitro* test system described previously (Gabbasov *et al*. 2021). Briefly, whole blood was perfused over a fibrinogen-coated surface at the shear rate of 1300 s^−1^ for 15 min, and platelet adhesion was registered in real time by detection of scattered laser light. Blood samples were anticoagulated with D-phenylalanyl-L-prolyl-L-arginine chloromethylketone (PPACK) in a final concentration of 100 µmol/L. Prior to the perfusion experiment, 5 μmol/L ADP was added to achieve uniform activation of platelets in different samples. For each patient/volunteer, two separate measurements were performed: with and without the addition of a monoclonal antibody (mAb) against GPIb. Blood samples were preincubated for 3 min with 20 µg/mL of mAb AK2 generously gifted by Prof. A.V. Mazurov (Berndt *et al*. 1988). The difference in platelet adhesion between those two measurements was calculated in percent (%) from the level of adhesion observed without the addition of mAb.

**Figure 1:**
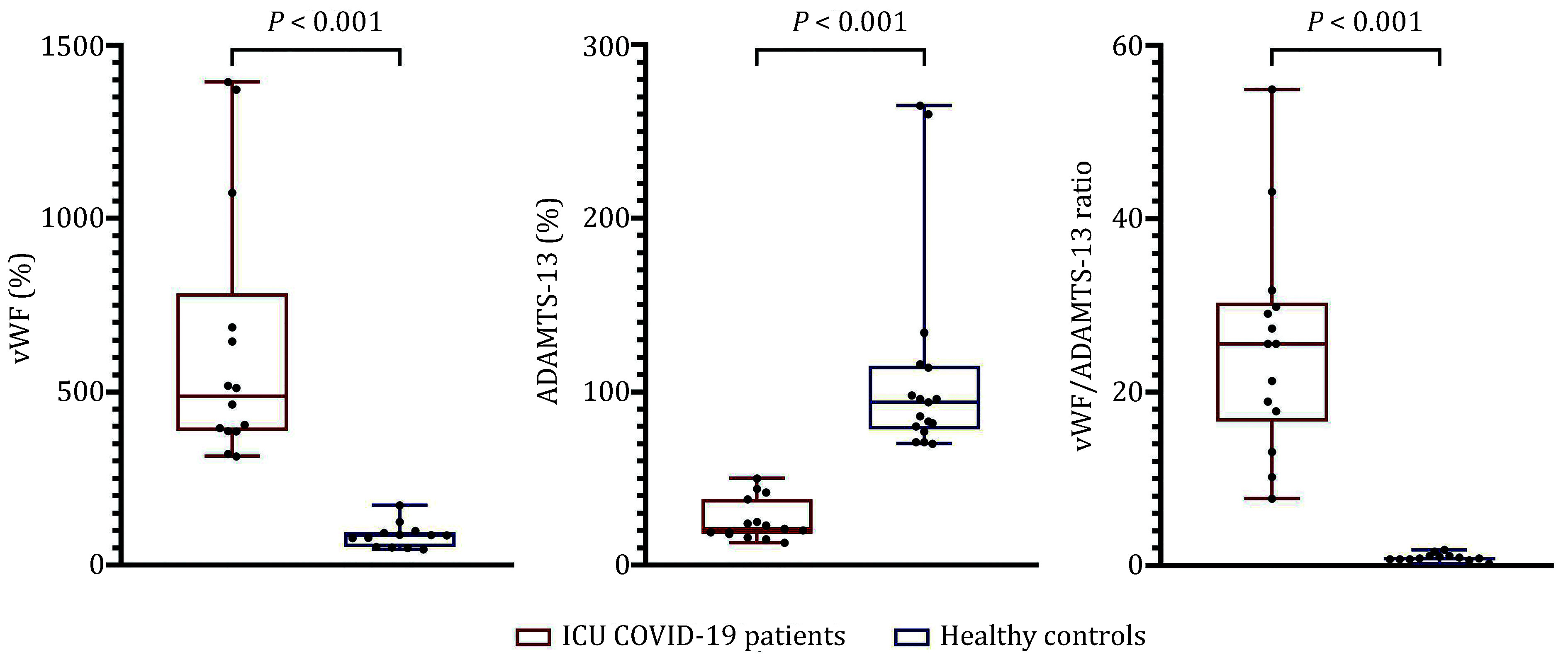
vWF levels and ADAMTS-13 activity in blood plasma, and vWF/ADAMTS-13 ratio of intensive care unit (ICU) COVID-19 patients and healthy controls; *P*, comparison of two independent groups (Mann-Whitney *U* test)

Patient characteristics are summarized in Table 1. It can be clearly seen that in our group of patients, the vWF/ADAMTS-13 axis was heavily imbalanced. We have observed a massive increase in vWF levels (median 488%, interquartile range (IQR) 389%–676%) and a pronounced deficiency of ADAMTS-13 activity (median 21%, IQR 18.5%–31.5%). These observations are in line with previous studies including ICU patients with COVID-19 (Favaloro *et al*. 2021). It is worth mentioning that ADAMTS-13 activity in all the patients was above the 10% level indicative of TTP. At the same time, the decrease of ADAMTS-13 activity was relatively high in comparison to other previously reported studies, which might be attributed to the severity of the illness in our cohort of ICU patients (Fig. 1). Raised levels of D-dimer and C-reactive protein were also observed (see Table 1).

**Table 1:** Characteristics & blood variables for ICU COVID-19 patients and healthy volunteers

Variable	ICU COVID-19 patients (*n* = 17)	Healthy controls (*n* = 17)	*P-*value
Age (years)	67 (62–71)	50 (44–57)	<0.01
Sex (F:M)	12:5	8:9	0.30
Platelets (1/nL)	208 (105–267)	166 (130–198)	0.82
Leukocytes (1/nL)	9.4 (5.4–11.5)	4.5 (4.0–6.3)	0.01
RBC (1/nL)	4.6 (3.9–5.0)	4.2 (4.1–4.6)	0.64
Hemoglobin (g/dL)	13.7 (12.1–14.2)	14.1 (13.3–15.5)	0.21
C-reactive protein (mg/L)	22.3 (6.7–66.9)	1.8 (0.4–2.6)	<0.01
Procalcitonin (ng/mL)	0.08 (0.04–0.17)	0.06 (0.03–0.08)	0.29
Fibrinogen (g/L)	3.4 (2.7–4.8)	3.1 (2.9–3.9)	0.87
D-dimer (ng/mL)	852 (598–2196)	110 (7–290)	<0.01
APTT (s)	27 (23–32)	29 (27–33)	0.34
vWF (%)	488 (389–676)	86 (53–93)	<0.01
ADAMTS-13 (%)	21 (19–32)	94 (80–114)	<0.01
Results of platelet adhesion measurements			
Adhesion without mAb (a.u.)	42.1 (23.0–70.3)	40.7 (31.9–56.9)	0.73
Adhesion with mAb (a.u.)	9.4 (5.2–25.0)	29.1 (13.1–47.6)	0.03
Inhibition (%)	63.9 (47.5–83.8)	45.0 (20.7–49.7)	<0.01
Note: Continuous data expressed as median and interquartile range (between brackets). *P-*values of the Mann-Whitney test are shown. Abbreviations: F, female; M, male; APTT, activated partial thromboplastin time; RBC, red blood cell count; vWF, von Willebrand factor; ADAMTS-13, a disintegrin and metalloproteinase with thrombospondin motif 13			

**Figure 2:**
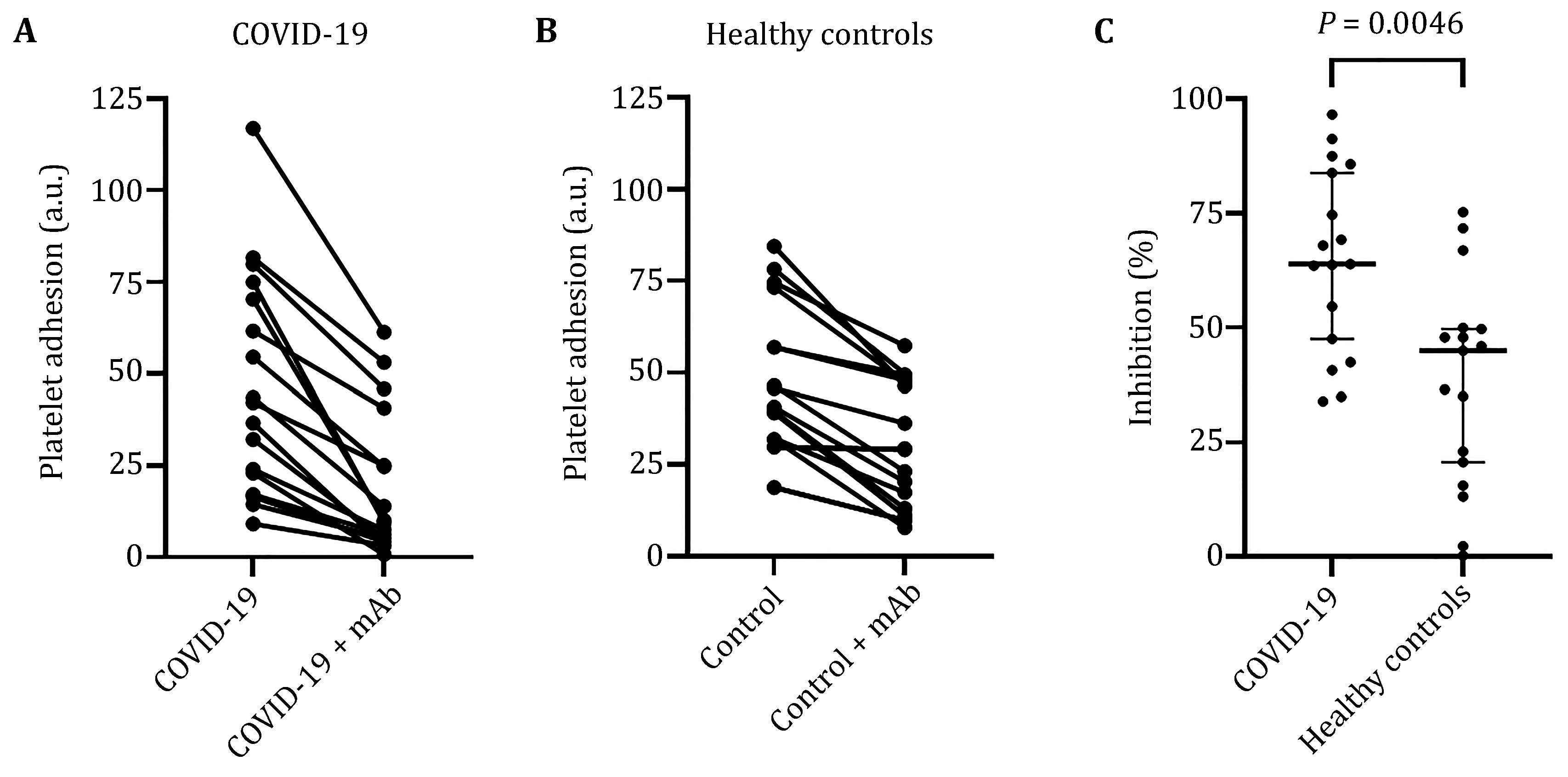
Platelet adhesion and its inhibition by anti-GPIb mAb. **A**,**B** Platelet adhesion for ICU COVID-19 patients (*n* = 17) and healthy controls (*n* = 17), with and without addition of mAb blocking GPIb. Wilcoxon matched-pairs signed-rank test *P* < 0.0001. **C** Inhibition of platelet adhesion by anti-GPIb antibody calculated as percent of adhesion in experiments without addition of inhibitor. Horizontal bars are medians and vertical bars interquartile range. *P-*value of the Mann-Whitney *U* test is shown

Results of platelet adhesion measurements are shown in Figs. 2A and 2B. No difference was observed in platelet adhesion between the COVID group and the non-COVID group (median 42.1 arbitrary units (a.u.) (IQR 23.0–70.3) vs 40.7 a.u. (IQR 31.9–56.9), *P* = 0.73, Mann-Whitney *U* test). In experiments with the addition of anti-GPIb mAb, a reduced platelet adhesion was observed in both groups: 9.4 a.u. (IQR 5.2–25.0) in the COVID-19 group and 29.1 a.u. (IQR 13.1–47.6) in the control group. Inhibition of platelet adhesion by anti-GPIb mAb was much more pronounced in the COVID-19 group (Fig. 2C). Relative inhibition of platelet adhesion by anti-GPIb mAb was 64% (IQR 48%–84%) and 45% (IQR 21%–50%) in COVID-19 and control groups, correspondingly (*P* = 0.0046, Mann-Whitney *U* test). In other words, the influence of GPIb on platelet adhesion was increased in severe COVID-19 patients in comparison to healthy controls.

Interestingly, an excess of vWF in severe COVID-19 patients did not lead to enhanced platelet adhesion in our experiments. The question of why there is no increase in platelet adhesion while very high levels of vWF are present in severe COVID-19 patients remains unclear. This observation seems rather counterintuitive. At the same time, a similar result concerning platelet adhesion under flow in a cohort of severe COVID-19 patients was reported in a recent study (Tacquard *et al*. 2023). Though experimental conditions were somewhat different, they also observed no increase of vWF-mediated platelet adhesion under high shear rates and even reported a non-significant tendency towards reduced platelet adhesion.

One might assume that excess of vWF is compensated by a defective adhesive function of platelets from patients with severe COVID-19, for instance by a reduced expression of platelet receptors. For today the data available on this subject in the literature is somewhat contradictory. While indicate a reduction in the surface expression of GPIIb/IIIa and GPIb in severe COVID-19 platelets, an earlier work (Tacquard *et al*. 2023), on the contrary, states significantly higher expression of both GPIIb/IIIa and GPIb in COVID-19 patients. There is also a chance that platelet receptor expression depends on the severity of COVID-19. This subject needs to be thoroughly analyzed in future studies. It also should be taken into account that both our study and the French study (Tacquard *et al*. 2023) are limited by a small number of included patients.

There are several limitations of the present study that need to be mentioned. First, a significant limitation of our work is the limited cohort of patients, which was not age/sex-matched with the control group. Although the results obtained in our study were statistically significant, their generalization on the overall population may require further investigation in a broader cohort of patients. Also, we didn’t test the patients for specific variants of SARS-CoV-2. Our study was performed during the second wave of COVID-19, therefore we suppose that the patients had the original SARS CoV-2 variant. There is a possibility that various SARS-CoV-2 variants may differ in their influence on platelet adhesion. It would be interesting to analyze the impact of different variants of SARS-CoV-2 on the effects described, but this is beyond the scope of the present study.

It is also worth noting that the use of fibrinogen-coated slides as an adhesive substrate in our study was a forced measure due to our temporal constraints and technical limitations at the time of the study. To our knowledge, vWF itself does directly bind to fibrinogen. We assume that the initial stage of platelet adhesion in our experimental model is mediated through platelet–fibrinogen interactions. At the same time, further growth of the platelet aggregate on the adhesive surface is governed by the formation of vWF strands and their interactions with GPIb and other platelet receptors. That is why we consider our setup suitable for analysis of the impact of vWF and GPIb on platelet adhesion in flowing blood. Of course, it would be very interesting to compare our results to a more direct approach, for instance, the one using collagen/vWF-coated slides.

An interesting observation made in the present study is the increased effect of GPIb on platelet adhesion in severe COVID-19 patients. Inhibition of platelet adhesion by anti-GPIb mAb was much more pronounced in the COVID-19 group compared to healthy controls. To our knowledge, similar results have not been reported previously. Explanation of this effect will for sure require further in-depth investigation. The following hypotheses might be tentatively suggested. Firstly, one may suppose that platelets of COVID-19 patients have a shifted balance in the expression of different platelet receptors involved in platelet adhesion under shear flow. Relatively increased expression of GPIb could potentially explain the effect observed. However, a separate series of thorough experiments is required before we can state or disprove this, because, as we have already mentioned, the data available for today is somewhat contradictory (Bongiovanni *et al*. 2021; Tacquard *et al*. 2023). A second possible hypothesis is a higher occupancy of GPIb complex by ligands such as SARS-CoV-2 spike protein. Further research is needed to clarify the influence of competing ligands to GPIb on platelet adhesion in COVID-19.

It is also worth noting that GPIb is known to be a major factor in thromboinflammatory processes in different pathological conditions including, for example, stroke (Denorme *et al*. 2019). Moreover, leukocyte integrin Mac-1 (also known as CD11b/CD18) directly interacts with platelet receptor GPIb promoting thrombosis (Wang *et al*. 2017). A couple of most recent studies indicate upregulation of Mac-1 (Hottz *et al*. 2022; Rieder *et al*. 2023) and increase of circulating platelet-neutrophil complexes in COVID-19 (Rieder *et al*. 2023). Taken together, these data suggest that GPIb might play a significant role in COVID-19 pathogenesis.

In conclusion, this study demonstrates that the contribution of GPIb to platelet adhesion was significantly altered in patients with severe COVID-19. Further investigations are necessary to determine the pathogenic mechanisms that produced this change.
